# Prediction of Patient Outcomes in Locally Advanced Cervical Carcinoma Following Chemoradiotherapy—Comparative Effectiveness of Magnetic Resonance Imaging and 2-Deoxy-2-[^18^F]fluoro-D-glucose Imaging

**DOI:** 10.3390/cancers16030476

**Published:** 2024-01-23

**Authors:** Simran Singh Dhesi, Russell Frood, Sarah Swift, Rachel Cooper, Siddhant Muzumdar, Mehvish Jamal, Andrew Scarsbrook

**Affiliations:** 1 Department of Radiology, Leeds Teaching Hospitals NHS Trust, Leeds LS9 7TF, UK; s.dhesi2@nhs.net (S.S.D.); russellfrood@nhs.net (R.F.); sarah.swift1@nhs.net (S.S.); mehvish.jamal@nhs.net (M.J.); 2 Leeds Institute of Health Research, Faculty of Medicine & Health, University of Leeds, Leeds LS9 7TF, UK; 3 Department of Clinical Oncology, Leeds Cancer Centre, Leeds LS9 7TF, UK; rachel.cooper1@nhs.net; 4 Department of Radiology, Chelsea & Westminster Hospital, 369 Fulham Rd., London SW10 9NH, UK; siddhant.muzumdar@nhs.net

**Keywords:** PET-CT, MRI, diffusion-weighted imaging, cervical cancer, radiotherapy, chemotherapy, recurrence

## Abstract

**Simple Summary:**

This study investigates the effectiveness of three imaging methods—T2-weighted imaging (T2WI), diffusion-weighted imaging (DWI), and 2-deoxy-2-[^18^F]fluoro-D-glucose positron emission tomography-computed tomography (2-[^18^F]FDG PET-CT)—individually and combined, in assessing treatment response for locally advanced cervical carcinoma (LACC). As the third most common cancer worldwide, precise post-treatment evaluation is crucial for planning and follow-up. This research addresses the lack of a standardised response assessment after chemoradiotherapy for LACC, introducing a five-point qualitative scale for assessment. The findings aim to fill knowledge gaps in treatment response evaluation, potentially influencing clinical practices for better patient outcomes in cervical cancer management.

**Abstract:**

Purpose: To evaluate the utility and comparative effectiveness of three five-point qualitative scoring systems for assessing response on PET-CT and MRI imaging individually and in combination, following curative-intent chemoradiotherapy (CRT) in locally advanced cervical cancer (LACC). Their performance in the prediction of subsequent patient outcomes was also assessed; Methods: Ninety-seven patients with histologically confirmed LACC treated with CRT using standard institutional protocols at a single centre who underwent PET-CT and MRI at staging and post treatment were identified retrospectively from an institutional database. The post-CRT imaging studies were independently reviewed, and response assessed using five-point scoring tools for T2WI, DWI, and FDG PET-CT. Patient characteristics, staging, treatment, and follow-up details including progression-free survival (PFS) and overall survival (OS) outcomes were collected. To compare diagnostic performance metrics, a two-proportion z-test was employed. A Kaplan–Meier analysis (Mantel–Cox log-rank) was performed. Results: The T2WI (*p* < 0.00001, *p* < 0.00001) and DWI response scores (*p* < 0.00001, *p* = 0.0002) had higher specificity and accuracy than the PET-CT. The T2WI score had the highest positive predictive value (PPV), while the negative predictive value (NPV) was consistent across modalities. The combined MR scores maintained high NPV, PPV, specificity, and sensitivity, and the PET/MR consensus scores showed superior diagnostic accuracy and specificity compared to the PET-CT score alone (*p* = 0.02926, *p* = 0.0083). The Kaplan–Meier analysis revealed significant differences in the PFS based on the T2WI (*p* < 0.001), DWI (*p* < 0.001), combined MR (*p* = 0.003), and PET-CT/MR consensus scores (*p* < 0.001) and in the OS for the T2WI (*p* < 0.001), DWI (*p* < 0.001), and combined MR scores (*p* = 0.031) between responders and non-responders. Conclusion: Post-CRT response assessment using qualitative MR scoring and/or consensus PET-CT and MRI scoring was a better predictor of outcome compared to PET-CT assessment alone. This requires validation in a larger prospective study but offers the potential to help stratify patient follow-up in the future.

## 1. Introduction

Cervical carcinoma ranks as the third most prevalent malignancy globally [1,2], with up to 40% of women presenting with locally advanced cervical carcinoma (LACC) [3]. The age-standardised incidence of cervical cancer stands at 13.1 per 100,000 women, contributing over 300,000 deaths annually [4,5].

Accurate diagnosis and staging of LACC are vital for effective treatment planning. Current methods involve clinical history, pelvic examination, cystoscopy, biopsy, and colposcopy, with pelvic magnetic resonance imaging (MRI) recommended for an initial extent evaluation. Additionally, 2-deoxy-2-[^18^F]fluoro-D-glucose (2-[^18^F]FDG) positron emission tomography-computed tomography (PET-CT) is recommended for the confirmation of nodal disease and metastatic staging [6,7,8]. Recent guidelines advocate for the use of both TNM and FIGO systems, superseding reliance on the FIGO system alone [8].

Early-stage cervical cancer differs from LACC in terms of treatment approaches, with surgery preferred for the former and concurrent chemoradiotherapy (CRT) as the standard for the latter [8,9,10,11]. Image-guided adaptive brachytherapy (IGABT) is considered in most cases, provided the topography of the tumour allows for it, while (salvage) surgery is reserved for treatment failure or recurrence [9]. While more than 90% of patients with LACC achieve complete response to treatment initially, up to 33% of patients experience disease recurrence within two years post therapy [12,13]. Therefore, a precise response assessment is crucial for detecting residual tumour or recurrence, facilitating potential curative surgery [14].

The current response assessment post CRT in patients with LACC lacks standardisation, relying on a combination of imaging and clinical examination, which is hindered by anatomical changes and patient discomfort [14]. The principal imaging modalities for assessing tumour extent post CRT encompass MRI and 2-[^18^F]FDG PET-CT, each offering distinct advantages and disadvantages [15].

MRI is commonly used, but the interpretation of T2-weighted imaging (T2WI) can be challenging due to signal similarities between post-radiotherapy oedema and necrotic tumour, necessitating repeat imaging to avoid false positives [16].

Diffusion-weighted imaging (DWI) is increasingly incorporated into LACC MRI protocols to mitigate diagnostic uncertainty on T2WI. Apparent diffusion coefficient (ADC) values from DWI indicate tumour aggressiveness, but there is a lack of evidence regarding firm cut-off values or quantitative derivation that is clinically reproducible [16]. In contrast, qualitative analyses, such as the use of five-point ordinal scales, are now the standard-of-care for assessing treatment response post therapy in patients with lymphoma, with similar methods being used in head and neck oncologic imaging to stratify patient management post CRT [17,18,19]. There is no consensus on the use of a qualitative scale for assessing MRI response in patients with LACC.

Contrastingly, 2-[^18^F]FDG PET-CT excels in evaluating tumour metabolic activity compared to MRI, demonstrating a strong correlation between measured size on imaging and pathological specimen size [20]. PET-CT also plays a significant role in post-treatment surveillance with several single-centre studies that have demonstrated that 2-[^18^F]FDG PET-CT performed post CRT can independently predict patient outcomes in LACC [21,22,23,24]. The timely assessment provided by PET-CT allows for potential treatment modifications in cases of a demonstrated poor response either during or immediately post therapy [20]. Additionally, PET-CT proves valuable in the assessment of distant metastatic disease, including the involved lymph nodes, thereby enhancing any subsequent treatment [25]. Notable disadvantages, however, include the difficulty in the detection of low-volume recurrence or untreated disease, which may be masked by physiologic, non-pathologic, or inflammatory states, leading to increased metabolic activity and subsequent false positives [26,27].

The use of a five-point qualitative response assessment scale for 2-[^18^F]FDG PET-CT to predict outcomes after CRT in patients with LACC has been proposed, potentially enhancing subsequent patient treatment even further and facilitating more personalised risk-adapted follow-ups in line with recent European Society of Gynaecological Oncology (ESGO) guidance [8,22].

Despite these developments, the comparative effectiveness of MRI and 2-[^18^F]FDG PET-CT for treatment response evaluation in patients with LACC after non-surgical treatment remains an under-researched topic [28,29]. Moreover, investigation into the utilisation of combination assessment is warranted, especially in light of promising and favourable outcomes observed in previous studies involving biologically similar cancers, such as anal cell carcinoma [30]. This could be used to offer personalised follow-ups, potentially alleviating the substantial reduction in the quality of life of and economic impact to the patient associated with both the disease and its subsequent follow-up [31].

Therefore, the primary aim of this study was to evaluate the utility and comparative effectiveness of three five-point qualitative scoring systems for the evaluation of response in 2-[^18^F]FDG PET-CT and MRI imaging, both individually and in tandem, following curative-intent CRT in patients with LACC. The secondary aim was to assess the performance of 2-[^18^F]FDG PET-CT and MR in the prediction of subsequent patient outcomes.

## 2. Materials and Methods

### 2.1. Ethics Approval

This study has been developed upon the informed consent of the patients undergoing the diagnostic procedures for the imaging studies and uses their anonymised personal data for scientific research purposes. We obtained formal approval from the Ethics Committee to use radiological imaging and clinical data including retrospective retrieval of anonymised patient data from institutional databases (RCD-Onc: Enhancing understanding and prediction of cancer outcomes with baseline characteristics from routinely collected data, Integrated Research Application Approval Number 277122).

### 2.2. Study Population and Follow-Up

A retrospective analysis of the data was conducted from consecutive patients with histologically confirmed LACC treated between June 2014 and December 2021 at a single tertiary referral centre. Baseline and response assessment MRI and 2-[^18^F]FDG PET-CT were undertaken, with the latter being performed approximately three months post CRT. Additional MRI assessments occurred at 6 and/or 12 months post treatment in the majority of patients. The inclusion criteria included patients with locally advanced cervical cancer receiving initial curative-intent CRT treatment only, in accordance with departmental protocols. The exclusion criteria included patients with metastatic disease outside of the radiation therapy volume, those with surgically resected cancer, those who had undergone previous CRT for LACC, or those deemed ineligible for curative-intent CRT.

The patients were followed up with a physical examination consisting of an abdominal and gynaecological exam 6 weeks post CRT. For the first two years post treatment, the patients were followed up in three-monthly intervals where follow-up included a physical examination. This was supplemented by 2-[^18^F]FDG PET-CT at 3 months and MRI scans at 3 and 6 months post treatment. True complete responses as defined by a normal clinical examination and locoregional control at 3 months, received twelve-monthly rather than six-monthly MRI follow-ups.

Data, including clinical history, patient demographics, tumour staging, treatment, and progression-free survival [PFS] and overall survival [OS] outcomes, were obtained from the institutional electronic patient record system (PPM+, Leeds, UK). The subject characteristics of the patients including their histological sub-groups are provided in Table 1. PFS was defined as LACC-related death, time from treatment completion to locoregional failure, or new distant metastatic disease. OS, on the other hand, was defined as the time from completion of CRT to death, regardless of the cause.

### 2.3. Concurrent Chemoradiotherapy Regimen

The patients underwent either pelvic three-dimensional conformal external beam radiotherapy (EBRT) or volumetric modulated arc therapy (IMRT) concurrently with chemotherapy. The prescribed doses were 48 Gy in 24 fractions for EBRT and 45 Gy in 25 fractions for IMRT. For those receiving the IMRT protocol, a simultaneous integrated boost of 55–57.5 Gy was prescribed specifically for large lymphadenopathy (short axis > 1 cm). The overall dose for both EBRT and IMRT remained consistent, ensuring an equivalent dose in two Gy/fraction (EQD2) of >85 Gy to a high-risk clinical target volume. In addition to radiotherapy, the majority of the patients received concurrent IV cisplatin chemotherapy (40 mg/m^2^). Exceptions were noted for a few patients whose age, performance status, or comorbidities necessitated the use of alternative chemotherapy agents. Post CRT, the majority of the patients received a high-dose-rate intra-cavity brachytherapy (BRT) boost, delivered in three fractions over three weeks, typically within 10 days of completing their CRT regimen. The dosing adhered to International Commission on Radiation Units and Measurements Reports 62 and 38 guidelines [32] and institutional treatment guidelines.

### 2.4. Image Acquisition

Standardised departmental protocols were employed for all PET-CT studies performed using two GE Healthcare Discovery 690 and 710 scanners (GE Healthcare, Madison, WI, USA). The use of multiple scanners followed the standard procedure in our Trust. It was anticipated that any potential discrepancies in the results stemming from inter-scanner variability would be negligible. CT was employed for localising anatomy and correcting for attenuation. Serum blood glucose levels were routinely assessed before the scan, with image acquisition deferred if the levels exceeded 10 mmol/L. The patients adhered to a 6 h fasting period before receiving 4 MBq/kg of 2-[^18^F]FDG via intravenous injection. Imaging was conducted 60 min after tracer injection without administration of iodinated contrast media. The imaged volumes were from the skull base to the upper thighs.

The study reformats comprised the following:

1. Non-contrast 2.5 mm thick axial reformats with applied soft tissue kernel, extending from the skull base to the proximal femora.
2. Non-contrast 2.5 mm thick axial reformats with applied lung kernel, extending from the lung apices to the upper abdomen.
3. Non-attenuation-corrected axial reconstructions of 2-[^18^F]FDG activity extending from the skull base to the proximal femora.
4. Maximum intensity projection (MIP) 3D reconstruction of FDG activity.
5. Fused 2-[^18^F]FDG/CT axial reconstructions with slice thickness of 2.5 mm.
6. Fused 2-[^18^F]FDG/CT coronal reconstructions with slice thickness of 2.5 mm.

Baseline and follow-up pelvic MRI was conducted using standardised departmental protocols on a 1.5-T Siemens Aera scanner (Siemens Healthcare, Erlangen, Germany) with a body and pelvic phased-array coil. The study sequences comprised the following:

1. High-resolution small-field-of-view sagittal and axial T2-weighted (T2W) sequences (3 mm slice thickness).
2. T2-weighted gradient echo images, including axial, coronal, and oblique reformats (3 mm slice thickness).
3. Diffusion-weighted image (DWI) sequences (b0, b150, b500) in axial plane with a corresponding apparent diffusion coefficient (ADC) map (4 mm slice thickness).
4. Multi-shot turbo spin echo (TSE) T2 transverse and T1 coronal plane sequences (balanced steady state free precession line acquisition with undersampling, BLADE) (10 mm and 4 mm slice thicknesses, respectively).

The patients fasted for 6 h before the examination and arrived with full bladders to minimise bowel motion artefact and improve their assessment.

### 2.5. Image Analysis

MRI interpretation utilised the picture-archiving and communication system (PACS)—Impax^®^ PACS v.5.6, (Agfa HealthCare, Mortsel, Belgium), while PET-CT interpretation was carried out on a workstation employing Advantage Windows, v.3.5 (GE Healthcare, Madison, WI, USA). A clinician with 2 years of experience in clinical medicine, supervised by a dual-certified radiologist/nuclear medicine physician and a radiologist with a special interest in gynaecological imaging with over 15 years of experience in PET-CT and MRI, respectively. The post-CRT imaging was compared with the baseline studies.

Five-point scales were employed to score the patients for residual tumoral signal on T2WI and DWI as well as for 2-[^18^F]FDG activity on the PET-CT. Interpretation was performed on a patient-by-patient basis, and any discrepancies were resolved through consensus agreement.

### 2.6. PET-CT Criteria

**Figure 1:**
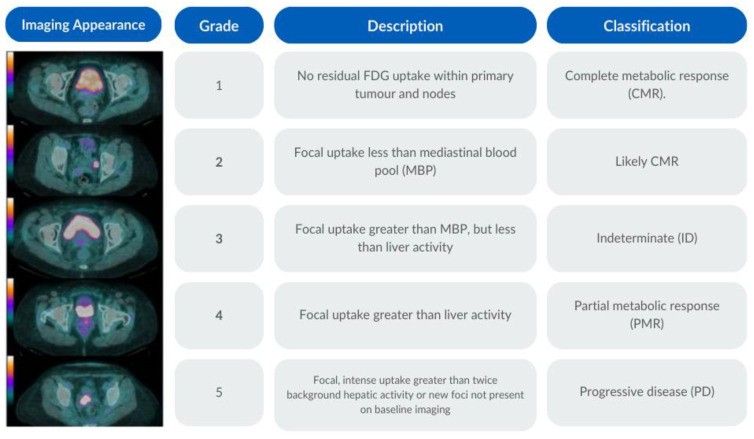
2-[^18^F]FDG PET-CT response assessment scale.

No residual 2-[^18^F]FDG uptake within the primary tumour and nodes was classified as a complete metabolic response (CMR) (grade 1); a focal uptake less than the mediastinal blood pool (MBP) was classified as a likely complete metabolic response (grade 2); a focal uptake greater than the mediastinal blood pool but less than the liver activity was classified as indeterminate (ID) (grade 3); a focal update greater than the liver activity was classified as a partial metabolic response (PMR) (grade 4); a focal intense uptake greater than twice the background hepatic activity or new foci not present in the baseline imaging were classified as progressive disease (PD) (grade 5). The 2-[^18^F]FDG PET-CT response assessment scale with the associated imaging appearance is documented in Figure 1.

### 2.7. MRI Criteria

**Figure 2:**
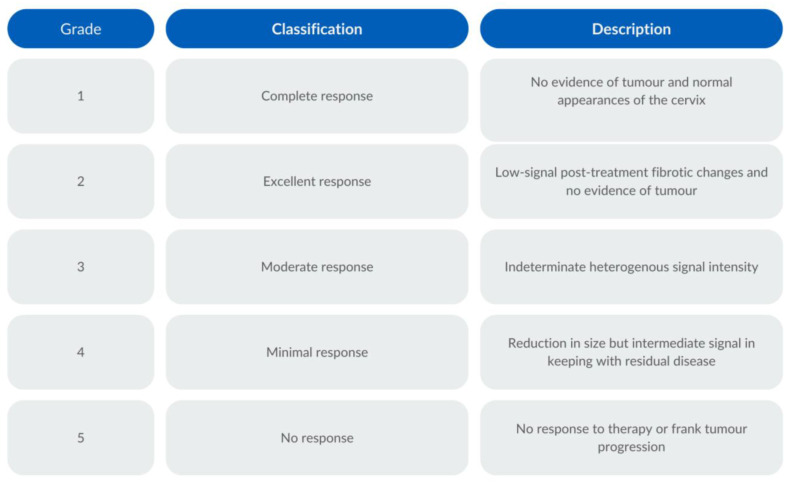
T2-weighted imaging response assessment scale.

**Figure 3:**
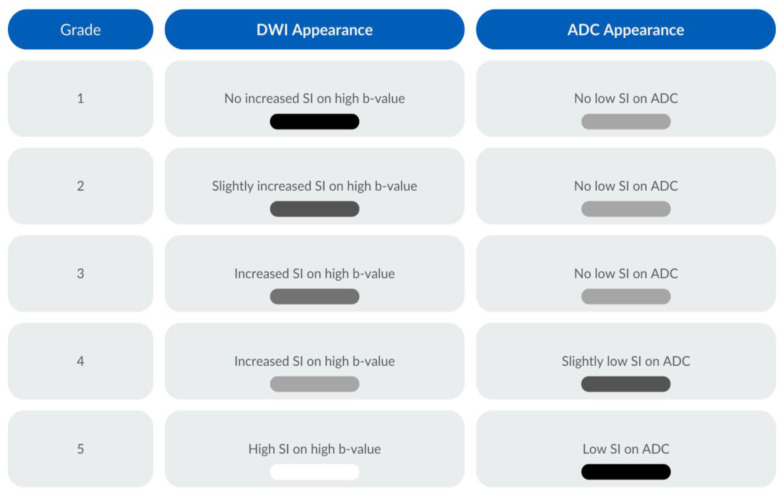
Diffusion-weighted imaging response assessment scale. The grayscale colours refer to the expected signal intensity on the DWI and the corresponding ADC map.

Tumour response assessment on T2WI was evaluated through the examination of morphological features and signal intensities within the cervix and/or nodal or metastatic disease in the imaged volume. A complete response (grade 1) signified the absence of discernible tumour/nodal or metastatic disease, with restoration of a normal cervical appearance. An excellent response (grade 2) was determined by the presence of a low signal intensity indicative of post-treatment fibrotic changes, with no observable tumour, nodal, or metastatic remnants. A moderate response (grade 3) was defined by a heterogeneous signal intensity and an indeterminate appearance of the cervix. A minimal response (grade 4) was denoted by a reduction in tumour size but a persistent intermediate signal intensity, suggesting the presence of residual disease. No response (grade 5) was determined where no therapeutic effect was evident or the tumour exhibited frank progression and/or new nodal/metastatic disease. Tumour response assessment was carried out based on the highest b-value for the DWI and the accompanying ADC map, with the following grading system: no increased signal intensity (SI) on the DWI and no low SI on the ADC were considered a grade 1 response; a slightly increased SI on the DWI and no low SI on the ADC were considered to be a grade 2 response; an increased SI on the DWI and no low SI on the ADC were categorised as a grade 3 response; an increased SI on the DWI and a slightly low SI on the ADC constituted a grade 4 response; and a high SI on the DWI and a low SI on the ADC were a grade 5 response. The T2-weighted and DWI response assessments are illustrated in Figure 2 and Figure 3, respectively.

In order to reference the assessments against clinical outcomes, receiver operating characteristic (ROC) curves were created for each imaging modality to determine the optimal classification threshold for treatment response. A literature search was conducted, which identified the consensus criteria used to combine T2WI and DWI as well as those for reaching a consensus for MR and PET-CT parameters [30,33].

### 2.8. Statistical Analysis

Statistical analysis was conducted using Python (v. 3.9.6) along with the NumPy (v. 1.2.5) and Pandas (2.1.1) libraries. A two-proportion z-test for comparing specificity, sensitivity, positive predictive value (PPV), negative predictive value (NPV), and accuracy was grounded in its appropriateness for assessing differences in proportions between two binomial and independent groups [34]. Statistical significance was determined with a two-tailed *p*-value of <0.05.

A time-to-event analysis with a starting point designated as CRT completion was conducted. Kaplan–Meier survival plots were generated and an analysis using the Mantel–Cox log-rank test was performed using the Python library KaplanMeierFitter, part of lifelines (v. 0.27.8). The use of Kaplan–Meier survival plots and the Mantel–Cox log-rank test is well-established for comparing survival distributions of censored data and is suited for assessing treatment outcomes. The log-rank test is often employed, owing to the rightward skew and censoring of data [35]. Our analysis also assessed PFS and OS.

The Cox proportional hazards model was employed to calculate hazard ratios (HR). This model was selected for its suitability in analysing time-to-event data for binary covariates [36].

## 3. Results

### 3.1. Study Group

Ninety-seven patients, with a median age of 47 years (range: 24–82), were analysed. The histological subtypes included squamous cell carcinoma (79%), adenocarcinoma (15%), adenosquamous carcinoma (4%), and others (2%). Forty-five patients had nodal disease at the baseline, and the follow-up continued until death or the 1 July 2023, with a median of 49.5 months (range: 2.3–90.6 months). See Table 1 for the subject characteristics, including the histological sub-groups.

### 3.2. Overall and Progression-Free Survival

Twenty-three out of the ninety-seven (24%) subjects demonstrated residual cervical, nodal, or metastatic disease after CRT, defined clinically by outcome. Of these subjects, fifteen demonstrated persistent cervical disease, nine with interval progression in their primary tumour and six with persistent cervical/pelvic nodal disease. Five subjects demonstrated locoregional metastatic disease, with a further three patients who developed new systemic metastatic disease found during our response assessment evaluation. During the follow-up period, twenty-seven subjects (28%) died owing to disease progression. The median time to progression was 166 days (range 72–1395 days), and the median time to death was 706 days (range 90–1834 days).

### 3.3. Imaging Analysis

#### 3.3.1. Image Grouping

**Figure 4:**
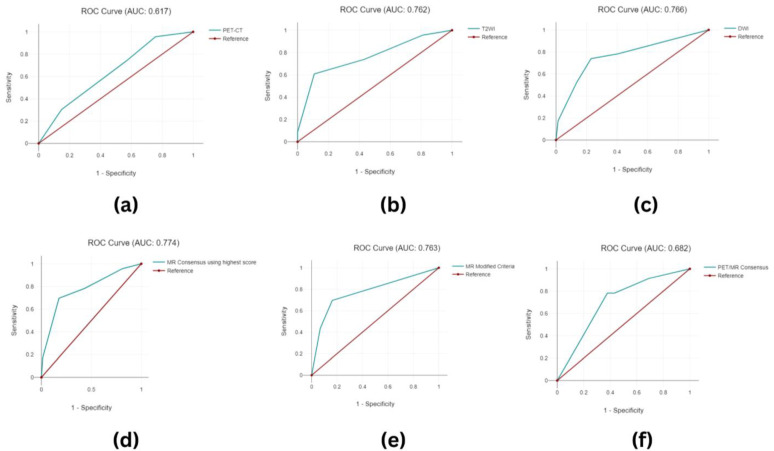
Region under the curve (ROC) graphs for (**a**) PET-CT, (**b**) T2WI, (**c**) DWI, and (**d**) MR consensus using the highest score criteria, for (**e**) MR consensus using the modified criteria, and for (**f**) PET/MR consensus.

A threshold value of four or greater for the PET-CT and T2WI and three or greater for the DWI signified an incomplete response. A modified version of the consensus criteria for the T2WI and DWI was proposed and is detailed in Table 2. This was compared against using the highest score for either parameter. The respective ROC curves demonstrated a marginally better area under the curve (AUC) (77.4) for the highest score method when compared to the modified criteria (AUC 76.3). Therefore, the former method was used for MR harmonisation. A modified version of the criteria for the harmonisation of consensus MR and PET-CT was used and is detailed in Supplemental Table S1. The ROC curve for data harmonisation using the highest score for MR consensus and the modified criteria demonstrated an AUC of 68.2. The associated ROC curves and diagnostic performance tables for the PET-CT, T2WI, DWI, and combined data are shown in Figure 4 and Table 3, respectively.

#### 3.3.2. Image Classification

When considered in isolation, PET-CT, T2WI, and DWI demonstrated complete responses in 38 patients (39.2%), 75 patients (77.3%), and 63 patients (64.9%), respectively. PET-CT and DWI with sensitivities of 73.91% outperformed T2WI (60.87%), although this did not reach a statistical significance (PET-CT vs. T2WI *p* = 0.05238). Conversely, T2WI and DWI exhibited superior specificity and diagnostic accuracy when compared with PET-CT (Specificity: PET-CT 43.24%, T2WI 89.19%, and DWI 77.03% [PET-CT vs. T2WI *p* < 0.00001], [PET-CT vs. DWI *p* < 0.00001]. Accuracy: PET-CT 50.52%, T2WI 82.47%, and DWI 76.29% [PET-CT vs. T2WI *p* < 0.00001], [PET-CT vs. DWI *p* = 0.0002]). PPV was greatest for T2WI (89.19%), followed by DWI (50.00%), significantly outperforming PET-CT (28.81%) ([PET-CT vs. T2WI *p* < 0.00001], [PET-CT vs. DWI *p* = 0.00252]), whilst NPV was similar for all the parameters (PET-CT 84.21%, T2WI 88.00%, and DWI 90.48%). When combined, the MR consensus data maintained high NPV (89.71%), PPV (55.27%), specificity (82.43%), and sensitivity (69.67%). The PET/MR consensus had the highest diagnostic sensitivity (78.26%) when compared to all the other parameters and maintained NPV at 90.20%, with improvements in accuracy (65.98%), PPV (39.13%), and specificity (62.16%) when compared to the PET-CT alone (PET-CT/MR consensus vs. PET-CT *p* = 0.02926, *p* = 0.12852, and *p* = 0.0083, respectively) but not to the combined MR or individual T2WI and DWI.

**Figure 5:**
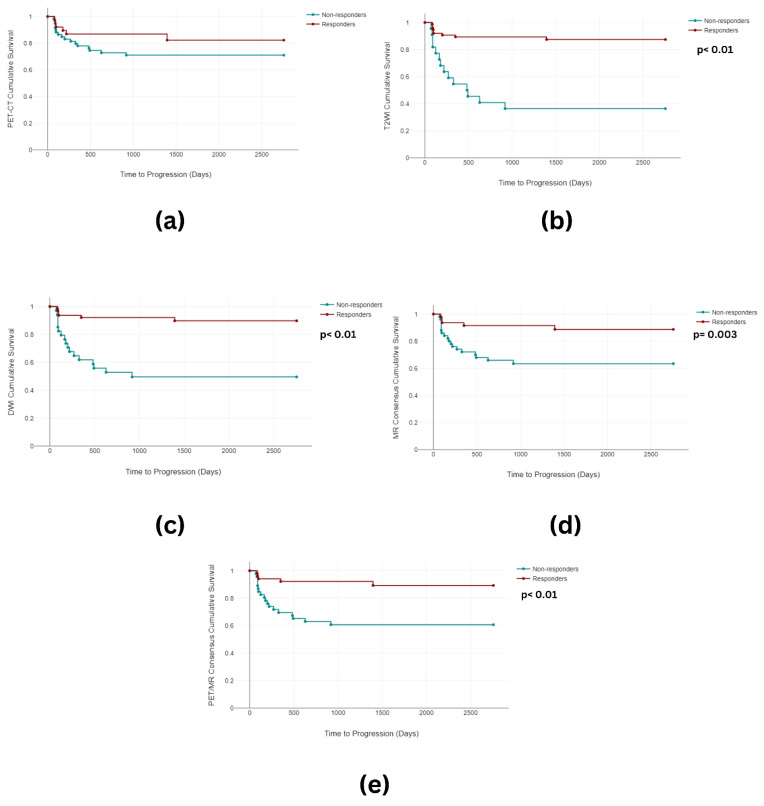
Kaplan–Meier curves depicting time to progression for (**a**) PET-CT, (**b**) T2WI, (**c**) DWI, (**d**) MR consensus, and (**e**) PET/MR consensus.

**Figure 6:**
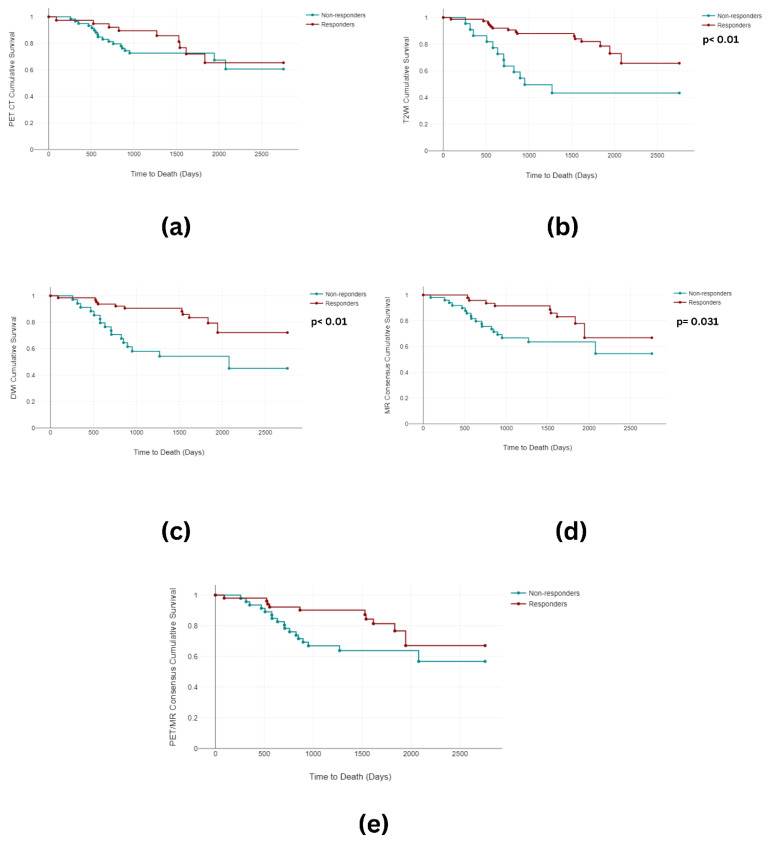
Kaplan–Meier curves depicting the time to death for (**a**) PET-CT, (**b**) T2WI, (**c**) DWI, (**d**) MR consensus, and (**e**) PET/MR consensus.

When examining the Kaplan–Meier analysis, statistically significant differences in the PFS between the responders (mean 1261 days) and the non-responders arose for T2W (mean 523 days) (*p* < 0.001), DWI (*p* < 0.001) (mean 1296 days vs. mean 592 days), the MR consensus (*p* = 0.003) (mean 1290 days vs. mean 680 days), and the PET-CT/MR consensus (*p* < 0.001) (mean 1298 days vs. mean 660 days). No significant differences were observed in the PET-CT PFS (Figure 5). Furthermore, differences reaching statistical significance were observed in the OS between the responders (mean 1851 days) and the non-responders for T2WI (mean 940 days) (*p* < 0.001), DWI (*p* < 0.001) (mean 1781 days vs. mean 1435 days), and the MR consensus (*p* = 0.031) (mean 1799 days vs. mean 1555 days). There was no significant difference in the OS between the responders (mean 1638 days) and the non-responders for PET-CT (mean 1670 days) (*p* = 0.489) or the PET-CT/MR consensus (*p* = 0.05) (mean 1766 days vs. mean 1573 days) (Figure 6).

The Cox regression analysis pertaining to the PFS for MR consensus findings showed a lower hazard ratio (HR) (HR = −1.03, *p* = 0.696) in comparison to the T2WI (HR = 1.17, *p* = 0.054) and DWI (HR = 1.5, *p* = 0.169) alone, although this did not reach a statistical significance. Similarly, the HR values for PET-CT (HR = −0.22, *p* = 0.696) and the PET-CT/MR consensus (HR = −0.74, *p* = 0.358) also remained non-significant. In the context of the OS, the HR values for the MR consensus (HR = −0.35, *p* = 0.683) and the PET-CT/MR consensus (HR = 0.01, *p* = 0.986) were the lowest, yet similarly devoid of statistical significance (Table 4).

## 4. Discussion

This study demonstrates for the first time the use of response assessment scoring to evaluate individual and combined assessments of 2-[^18^F]FDG PET-CT, T2-weighted, and DWI in patients with LACC. In this study, we observed similar NPV across PET-CT, T2WI, and DWI, and these trends persisted when examining the results for consensus MR and combination PET/MR assessments. The sensitivity of PET-CT and DWI at 73.91% surpassed that of T2WI at 60.87%, reaching statistical significance. Additionally, T2WI and DWI exhibited superior specificity (T2WI: 89.19%, DWI: 50.00%) and diagnostic accuracy (T2WI: 82.47%, DWI: 76.29%) when compared with PET-CT (sensitivity 43.24%, accuracy 50.52%), also reaching statistical significance. Furthermore, the positive predictive value (PPV) for T2W (63.64%) and DWI (50.00%) significantly outperformed PET-CT (28.81%).

The existing literature supports the well-defined contributions of MRI and FDG PET-CT in the pre-treatment assessment of patients with LACC. MRI emerges as the preferred choice for evaluating parametrial, vaginal, cervical, bladder, and rectal involvement, as evidenced by previous studies [37,38]. On the other hand, PET-CT excels in sensitivity for detecting lymph node metastases and assessing involvement in distant sites such as the pelvic, para-aortic, inguinal, and supraclavicular regions, as well as the peritoneum, mesentery, gastrointestinal tract, pleura, and mediastinum [39]. The distinct strengths of these imaging techniques position them as valuable tools in guiding treatment decisions, helping doctors determine whether surgery or CRT is the optimal course of action [40,41].

While prior research has explored the utility of PET-CT in appraising post-CRT response among patients with LACC [22,42,43,44], it is important to note that the existing evidence base comprises only a limited number of studies. Nevertheless, PET-CT is widely employed in guiding decisions on subsequent treatments [6,45]. Some researchers have advocated for the integration of standardised MRI and PET-CT response assessment scores and proposed the inclusion of DWI in MR protocols to enhance their specificity [22]. This confirms the need for comprehensive and evidence-based strategies in post-CRT response evaluations.

Gynaecological examinations post radiation therapy are challenging due to vaginal adhesions and fibrosis, hindering accurate visualisation of the cervix. Additionally, post-radiotherapy MRI may face challenges in accurately assessing response due to inflammation-induced hyperintensity on T2WI [46]. Similarly, PET-CT evaluation can be influenced by inflammation and necrosis induced by radiotherapy [44]. Our study contributes to this discourse by exploring the use of an individual five-point qualitative scale for PET-CT, T2WI, and DWI for predicting tumour response in patients with LACC post CRT.

The examination of the PET-CT findings revealed a PPV and NPV of 28.81% and 84.21%, respectively. The PPV figures were lower than those reported in other published series (median 58.9%) [22,24,47,48,49]. These studies were conducted at variable time-points after CRT (median 2 months, range 1–5), potentially contributing to the observed range in the reported PPV values, and were few in number. Furthermore, the accurate assessment of post-treatment responses may have been hindered by the presence of prominent physiological tracer activity or inflammatory changes, with the absence of an established optimal time-point for re-evaluation being a further complicating factor. Our reported NPV findings aligned with figures demonstrated in the established literature.

Consensus MR demonstrated a PPV and NPV of 50% and 90.48%, respectively—figures comparable to those reported in the limited number of studies with published data [50,51] (PPV range 15–100%, NPV range 78.9–96%). These single-centre studies, involving 41 and 52 patients, respectively, again demonstrated a wide range of PPV values, likely due to their small sample size. When the specificity and accuracy of the consensus MR data were examined, the figures in our study outperformed those of the PET-CT alone, yielding highly significant differences in specificity (*p* = 0.02926) and accuracy (*p* = 0.0083). This may be secondary to the incorporation of DWI sequences, which help one mitigate diagnostic uncertainty compared to using T2WI alone [16]. This suggests that MR assessment may be better suited to the detection of residual disease and recurrence, although limitations in our study size preclude drawing definitive conclusions.

When assessing the findings from the PET-CT and MRI in combination, our study demonstrated some advantages compared to the PET CT findings alone, with statistically significant improvements in accuracy and specificity. In contrast, when compared to the combined MR findings, the PET/MR combination assessment exhibited a poorer performance in terms of specificity, PPV, and accuracy, while displaying an overall similar performance in sensitivity and NPV. In prior studies which examined response assessments post CRT for anal cancer, a malignancy sharing biological characteristics with cervical cancer, combination assessments of PET-CT and MRI results demonstrated a substantial advantage. Significant enhancements in false positives, true negatives, sensitivity, specificity, PPV, and overall accuracy were observed when compared to relying solely on MRI interpretation [30]. Therefore, whilst our findings suggest that a combination assessment in LACC cases is weaker regarding the former metrics, these observations could also be biassed by the relatively small sample size and/or choice of methodology for harmonising the data. Exploring alternative options for data harmonisation could prove valuable in determining whether such modifications could enhance the diagnostic performance of combined PET/MR consensus.

When examining survival, the log-rank Mantel–Cox test demonstrated significant associations in PFS prediction for T2WI, DWI, and MR and PET/MR consensus. Similarly, when OS was considered, statistically significant differences for T2WI, DWI, and MR consensus were observed, with PET/MR consensus lying at the threshold of significance (*p* = 0.05). Interestingly, the PET-CT findings did not reach significance for either PFS or OS in this study, despite previous single-centre studies on LACC which reported OS rates in patients with CR on PET-CT of around 90% at the 5 year mark and 95% at the 2 year mark, with 95–100% NPV [22] and a very low rate of asymptomatic recurrence (1.6% in cervical cancer patients) [42]. The hazard ratios for all imaging modalities except for PFS in PET-CT crossed the threshold of unity, with no statistically significant observations when examining either PFS or OS through individual or combined assessment metrics. Whilst the absence of significance may be attributed to the fact that no single imaging modality serves as a definitive predictive marker for outcomes, other plausible contributors, such as the limited sample size introducing variability and compromising statistical power or an insufficient follow-up time, may also be responsible.

In considering future research directions, the recent literature exploring the use of neoadjuvant chemo-immunotherapy in locally advanced cervical cancer (LACC) has demonstrated promising antitumor activity and a manageable adverse event profile [52]. The combination of neoadjuvant chemo-immunotherapy with radical surgery may herald a potential new treatment avenue in this population of patients. Assessing pre-treatment and post-therapeutic imaging appearances in this context may be vital for several reasons. Pre-treatment imaging could be potentially useful in stratifying patients for a neoadjuvant regimen, especially when considering that research has been limited to those individuals with mild–moderate risk factors for recurrence [52]. Post-therapeutic imaging, for example, with 2-[^18^F]FDG PET-CT and MRI, either individually or in combination, could risk-stratify patients who have shown an excellent response to neoadjuvant chemo-immunotherapy and may be crucial for informing decisions between surgery and surveillance as treatments evolve. This requires thorough investigation through future research. As has been alluded to, LACC is associated with significant effects on QoL and HRQoL post treatment [31]. The employment of a standardised follow-up, which may serve to provide personalised risk-adapted care, could help improve patients’ quality of life and reduce the economic burden associated with longer-term follow-up. Alternatively, the use of multiple diagnostic methods may increase patient burden, leading to the opposite of the intended effect. These are considerations that need to be taken into account when contemplating the direction of future research and the development of a standardised follow-up protocol.

Furthermore, the relationship between Human Papillomavirus (HPV) status and outcomes in squamous cell carcinoma across various organs, including the cervix, must be investigated in the broader context of pre- and post-imaging appearances [53]. Although the current study did not encompass an assessment of HPV status, its inclusion in subsequent research works stands as an avenue for inquiry. Qualitative assessments, including the application of five-point ordinal scales, have become the standard approach for evaluating treatment response after therapy in lymphoma cases. Comparable methodologies are employed in head and neck oncological imaging to stratify patient management following CRT [17,18,19]. Our study contributes to the ongoing discourse surrounding the challenges encountered in post-radiation therapy evaluations, shedding light on the limitations and strengths of 2-[^18^F]FDG PET-CT and MRI. The exploration of a five-point qualitative scale for each modality in predicting tumour response post CRT offers a valuable framework that could be adapted for assessing treatment response in other cancers that are biologically similar to cervical cancer, including those which may be linked to HPV infection, such as vulvar, vaginal, and penile cancers. Finally, ongoing investigations into Delta-radiomic features derived from 2-[^18^F]FDG PET-CT in patients with LACC post CRT have demonstrated promise for event-free survival prediction in preliminary work conducted within our research group [54]. These tentative findings require further validation through investigation with a larger sample size of patients.

This work presents the novel use of a five-point scoring tool for each modality and combination assessments in an initial single-centre study. This may limit the generalisability of the findings to a broader population. Being retrospective, the study design is inherently subject to biases associated with data collection over time, and future prospective evaluations may help reduce this bias. This study did not collate data related to the patients’ HPV status, an aspect which has been previously implicated as a causative agent in cervical carcinogenesis [53]. Data collection relating to HPV status and how this affects imaging appearances remains a direction for future research. The relatively small study size and the heterogeneity in the treatment approaches, including the exclusion of some patients from high-dose brachytherapy, were also factors that may have influenced the results. Finally, the scanning schedules of some patients were affected by the COVID-19 pandemic, introducing an external factor that might have influenced the timing and consistency of the imaging assessments. These limitations emphasise the need for cautious interpretation of the results and underscore the importance of future studies with larger sample sizes and prospective designs to validate our findings.

## 5. Conclusions

In conclusion, our study demonstrates that combining MR, PET-CT, and MRI for post-CRT response assessment in LACC cases yields more accurate predictive outcomes than using PET-CT alone. This multimodal approach could be a valuable clinical indicator for patient follow-up. However, independent validation is crucial before clinical translation of the proposed approach. Further studies with larger, multicentre, and prospective datasets are needed to ensure the reliability and generalisability of our findings.

## Figures and Tables

**Table 1:** Patient demographics.

Variables	n-97	No.
Age	Median	47
	Range	24–82
T stage	T1	7 (7%)
	T2	66 (68.0%)
	T3	19 (20%)
	T4	5 (5%)
Nodal disease at baseline	Yes	45
	No	52
Histological subtype	Squamous cell carcinoma	77 (79%)
	Adenocarcinoma	15 (15%)
	Adenosquamous carcinoma	3 (4%)
	Other	2 (2%)
Metastatic stage at baseline	M0	94
	M1	3
Primary tumour SUVmax	Median	15.1
	Range	4.7–52.5
Deaths		27
Progression		23
Follow-up period (days)	Median	1506
	Range	71–2757
Duration between treatment completion and MRI response assessment (days)	Median	92
	Range	48–238
Duration between treatment completion and PET-CT response assessment (days)	Median	95
	Range	16–246

**Table 2:** Modified scoring scheme adapted from Haider et al. (2006) [33].

Grade	T2W- + Diffusion-Weighted Imaging
0, Definitely not cancer	T2W = 1 or DWI = 1
1, Probably not cancer	T2W ≤ 3 and DWI = 2 or 3
2, Possible cancer	T2W ≥ 4 and DWI = 2 or 3 or T2W ≤ 3 and DWI = 4
3, Probably cancer	T2W < 4 and DWI = 4 or 5
4, Definite cancer	T2W ≥ 4 and DWI = 4 or 5

**Table 3:** Diagnostic performance tables for PET-CT, T2W, DWI, and combined data.

Response	Clinical Outcome	3m PET-CT Score	3m T2W Score	3m DWI Score	MR Consensus	PET/MR Consensus
Complete Response	74	38	75	63	68	51
Incomplete Response	23	59	24	34	25	46
False Positive		42	8	17	13	28
False Negative		6	9	6	7	5
True Positive		17	14	17	16	18
True Negative		32	66	57	61	46
Sensitivity		73.91%	60.87%	73.91%	69.57%	78.26%
Specificity		43.24%	89.19%	77.03%	82.43%	62.16%
Positive Predictive Value		28.81%	63.64%	50.00%	55.17%	39.13%
Negative Predictive Value		84.21%	88.00%	90.48%	89.71%	90.20%
Accuracy		50.52%	82.47%	76.29%	79.38%	65.98%

**Table 4:** Individual and combined modality evaluation of post-curative-intent chemoradiotherapy response in individuals with locally advanced cervical carcinoma.

(a)									
	Chi-Square				df				*p*-Value
**Progression-free survival analysis**									
PET-CT	1.94				1				0.164
T2WI	28.12				1				**<0.001**
DWI	21.19				1				**<0.001**
MR Consensus	8.85				1				**0.003**
PET/MR Consensus	11.36				1				**0.001**
**Overall Survival Analysis**									
PET-CT	0.48				1				0.489
T2WI	13.91				1				**<0.001**
DWI	11.08				1				**0.001**
MR Consensus	4.67				1				**0.031**
PET/MR Consensus	3.84				1				0.05
**(b)**									
	**Hazard Ratio**		**Lower 95% Confidence Interval**			**Upper 95% Confidence Interval**			***p*** **-Value**
**Progression-free survival analysis**									
PET-CT	−0.02		−1.34			0.89			0.696
T2WI	1.17		−0.02			2.37			0.054
DWI	1.5		−0.63			3.63			0.169
MR Consensus	−1.03		−3.36			1.3			0.385
PET/MR Consensus	0.74		−0.84			2.32			0.358
**Overall Survival Analysis**									
PET-CT	0.05		−0.90			1			0.913
T2WI	0.82		−0.27			1.91			0.14
DWI	0.98		−0.65			2.61			0.238
MR Consensus	−0.35		−2.03			1.34			0.683
PET/MR Consensus	0.01		−1.28			1.3			0.986

Bold denotes a statistically significant result.

## Data Availability

The data presented in this study are available on request from the corresponding author. The data are not publicly available due to institutional data-sharing restrictions.

## Supplementary Materials

The following supporting information can be downloaded at: https://www.mdpi.com/article/10.3390/cancers16030476/s1, Table S1: Modified scoring scheme used to harmonise consensus MR and PET-CT data. Adapted from Adusumilli et al. (2022) [30].
