# Supplementary material for: Prediction of Patient Outcomes in Locally Advanced Cervical Carcinoma Following Chemoradiotherapy—Comparative Effectiveness of Magnetic Resonance Imaging and 2-Deoxy-2-[18F]fluoro-D-glucose Imaging
Source: Cancers (Basel). 2024 Jan 23;16(3):476. doi: 10.3390/cancers16030476 (PMC10854890; doi:10.3390/cancers16030476)
Supplement: Supplementary file 1 [file cancers-16-00476-s001.zip › cancers-2805937-supplementary.pdf]

Supplemental Table S1. Modified scoring scheme used to harmonise consensus MR and PET-CT data. Adapted from Adusumilli et al. (2022) [30].

		PET-CT Grade				
		**1**	**2**	**3**	**4**	**5**
**MRI Grade**	**1**	1	1	1	1	5
	**2**	1	2	2	2	5
	**3**	1	2	3	4	5
	**4**	1	2	4	4	5
	**5**	5	5	5	5	5
